# A Clinical Prognostic Model Based on Preoperative Hematological and Clinical Parameters Predicts the Progression of Primary WHO Grade II Meningioma

**DOI:** 10.3389/fonc.2021.748586

**Published:** 2021-10-11

**Authors:** Peng Gao, Tengxiao Kong, Xuqiang Zhu, Yingwei Zhen, Hongjiang Li, Di Chen, Shanpeng Yuan, Dongtao Zhang, Henan Jiao, Xueyuan Li, Dongming Yan

**Affiliations:** ^1^ Department of Neurosurgery, The First Affiliated Hospital of ZhengZhou University, Henan, China; ^2^ Department of Neurosurgery, Shanghai Ninth People’s Hospital, Shanghai Jiao Tong University School of Medicine, Shanghai, China; ^3^ Department of Neurosurgery, The Second Affiliated Hospital of Zhengzhou University, Henan, China

**Keywords:** WHO grade II meningioma, hematological parameters, nomogram, inflammation, coagulation

## Abstract

**Purpose:**

The purpose was to explore the correlation between hematological parameters and the progression of WHO grade II meningioma, and establish a clinical prognostic model based on hematological parameters and clinical prognostic factors to predict the progression-free survival (PFS) of patients.

**Methods:**

A total of 274 patients with WHO grade II meningiomas were included. Patients were randomly divided into a training cohort (192, 70%) and a test cohort (82, 30%). In the training cohort, the least absolute shrinkage and selection operator Cox regression analysis were used to screen for hematological parameters with prognostic value, and the hematological risk model (HRM) was constructed based on these parameters; univariate and multivariate Cox regression analyses were utilized to screen for clinical prognostic factors, and a clinical prognostic model was constructed based on clinical prognostic factors and HRM. The prognostic stability and accuracy of the HRM and clinical prognostic model were verified in the test cohort. Subgroup analysis was performed according to the patients’ different clinical characteristics.

**Results:**

Preoperative neutrophil-to-lymphocyte ratio, lymphocyte-to-monocyte ratio, platelet-to-lymphocyte ratio, albumin-to-globulin ratio, D-dimer, fibrinogen, and lactate dehydrogenase were associated with the PFS of patients. The areas under curve of the HRM were 0.773 (95% confidence interval [CI] 0.707–0.839) and 0.745 (95% CI 0.637–0.852) in the training cohort and test cohort, respectively. The progression risk was higher in the high-risk group than that in the low-risk group categorized by the optimal cutoff value (2.05) of hematological risk scores. The HRM, age, tumor location, tumor size, peritumoral edema, extent of resection, Ki-67 index, and postoperative radiotherapy were the prognostic factors for the progression of meningiomas. The corrected C-index of the clinical prognosis model was 0.79 in the training cohort. Clinical decision analysis showed that the clinical prognostic model could be used to obtain favorable clinical benefits. In the subgroup analysis, the HRM displayed excellent prognostic stability and general applicability in different subgroups.

**Conclusions:**

Preoperative hematological parameters are associated with the postoperative progression of WHO grade II meningiomas. The clinical prognosis model constructed based on hematological parameters and clinical prognostic factors has favorable predictive accuracy and clinical benefits.

## Introduction

Meningioma, which originates from arachnoid cells, is the most common intracranial tumor, accounting for 38.3% of all primary intracranial tumors (1). Meningioma has been categorized into the three following histological grades by the World Health Organization (WHO): grade I/benign meningioma, grade II/atypical meningioma (AM), and grade III/malignant meningioma (2). AM is regarded as the transition stage between grade I/benign and grade III/malignant. Currently, surgical resection is regarded as the first-line treatment for AM, but the 5-year progression rate can reach 29–58% (3), and the progression-free survival (PFS) varies greatly among patients with AM. In recent decades, neurosurgeons have attempted to identify appropriate prognostic factors to classify the progression risk of patients with AM and to design subsequent treatment and follow-up protocols. Previous studies have demonstrated that several molecular markers, such as NF2, TERT, and H3K27me3, are associated with the prognosis of meningioma (4–6); however, the WHO has not explicitly classified these objective molecules as prognostic criteria in the latest guidelines (2). Most prognosis-related molecular parameters are obtained by gene sequencing. However, gene sequencing is too expensive to afford for most patients, which undoubtedly brings difficulties to the prognosis evaluation of patients. Therefore, a simple and reliable scoring system is needed to evaluate the progression risk and realize the hierarchical management of patients with AM.

Numerous studies have shown that the systemic inflammatory response and coagulation cascade play an important role in the origin, proliferation, and invasion of tumors (7–10). Chen et al. reported that preoperative neutrophil-to-lymphocyte ratio (NLR) and fibrinogen (FIB) were related to the PFS of patients with AM (11). Our previous study confirmed the prognostic value of lymphocyte-to-monocyte ratio (LMR), platelet-to-lymphocyte ratio (PLR), and albumin-to-globulin ratio (AGR) in glioma (12); other factors, such as D-dimer (DD), hemoglobin (HBG), lactate dehydrogenase (LDH), and red blood cell distribution width (RDW), have also been shown to be correlated with clinical outcomes in patients with glioma (13). However, it remains unclear whether these parameters have any prognostic value in AM. In this study, we explored the correlation between hematological parameters and the progression of AM, and established a clinical prognostic model based on hematological parameters and other clinical prognostic factors to predict the PFS of patients with AM. Our results could facilitate the development of hierarchical management protocols.

## Materials and Methods

### Study Population

We reviewed the clinical data of all patients with meningioma recorded in the central nervous system tumor database of the First Affiliated Hospital of Zhengzhou University, China, from January 2014 to June 2018, and screened patients according to the following inclusion criteria: (1) age ≥18 years; (2) preoperative Computed Tomography (CT) and Magnetic Resonance Imaging (MRI) showed intracranial lesions with imaging features of meningioma; (3) hematological data were complete; (4) pathological examination revealed a diagnosis of AM; and (5) the follow-up time exceeded 3 years and the data were complete. The exclusion criteria were as follows: (1) meningioma progression or multiple meningiomas; (2) other types of tumors; (3) had received drugs, surgery, or radiotherapy for tumor before admission; (4) a history of hematologic or autoimmune diseases; (5) the use of steroids, immuno-modulators, or anticoagulants before surgery; (6) liver or kidney dysfunction; and (7) local or systemic infection before surgery. The present study conforms to the guidelines issued in the Declaration of Helsinki and was approved by the Ethics Committee of the First Affiliated Hospital of Zhengzhou University (Approval Number: 2019-KY-176).

### Study Design

We recorded the basic clinical characteristics, hematological test results, imaging findings, pathological results, and follow-up results of 274 included patients. The Karnofsky Performance Status (KPS) score was used to assess the health status of patients (14). All patients were randomly divided into a training cohort (192, 70%) and a test cohort (82, 30%). In the training cohort, a hematological risk model (HRM) was constructed based on hematological parameters related to the prognosis of patients. Combined with the risk scores generated by the HRM and other clinical prognosis factors, the clinical prognosis model was established in the training cohort and validated in the test cohort.

### Pathological Examination

All tumor tissues were fixed immediately after surgical resection and submitted to the pathology department for hematoxylin-eosin staining and immunohistochemical analysis. AM was diagnosed according to the latest WHO guideline for Central Nervous System Tumors (2). According to the new guidelines, a pathologist re-evaluated pathological reports made before 2016, and the tissue paraffin was taken for re-staining and analysis if necessary. The Ki-67 index was recorded to assess the proliferative activity of tumor cells.

### Radiological Examination and Postoperative Radiotherapy

All patients included in this study underwent CT and contrast-enhanced MRI within 5 days before surgery, and some patients underwent Magnetic Resonance Spectroscopy when the diagnosis was difficult. The tumor location was evaluated using gadolinium-enhanced T1-weighted MRI, and skull base location and non-skull base location were defined as described previously (11). Tumor maximum size was also recorded at this time. Preoperative T2-weighted images and fluid-attenuated inversion recovery images were used to evaluate peritumoral brain edema (PTBE). Patients underwent contrast-enhanced MRI within 2 weeks after surgery. The extent of resection (EOR) was comprehensively evaluated by neurosurgeons based on the surgical records and postoperative MRI reports provided by radiologists. The EOR was divided into gross total resection (GTR) (Simpson grades I–II) and subtotal resection (STR) (Simpson grades III–V) (15). Skull invasion was considered when the preoperative CT showed that the inner plate of the skull was uneven or when the inner plate was found to be eroded by the tumor during the operation.

Within 2 weeks after surgery, the neurosurgeon decided on the postoperative radiotherapy (PORT) regimen based on the EOR and pathological results. If the AM were GTR but had a Ki-67 ≥10%, we recommended patients receive fractionated radiotherapy (51–54 Gy given in 1.7–1.8 Gy per fraction); in addition, we recommended that patients with AM STR receive fractionated radiotherapy (54–60 Gy given in 1.8–2.0 Gy per fraction).

### Hematological Test

Routine blood test, electrolyte, liver and kidney function, and coagulation function tests were examined within 5 days before surgery. We recorded neutrophil counts, lymphocyte counts, platelet counts, monocyte counts, HBG, RDW, LDH, FIB, DD, albumin, and globulin and calculated the NLR, LMR, PLR, and AGR.

### Follow-Up

All patients underwent contrast-enhanced MRI in the third month after surgery. The subsequent follow-up was carried out every 3 months for the first year after surgery and every 6 months from the second year after surgery. Patients who experienced any discomfort came to the hospital promptly. Progression of AM was considered in contrast-enhanced MRI that revealed new lesions or growing residual tumor during the follow-up period. The PFS was identified as the time from surgery to progression.

### Statistical Analysis

In all patients, the receiver operating characteristic (ROC) curve was utilized to calculate the optimal cutoff value of hematology parameters related to tumor progression (based on the Youden index). Hematological parameters were converted into dichotomized variables. If the actual value was higher than the optimal cutoff value, the score was 1; otherwise, it was 0. In the training cohort, univariate Cox regression and the least absolute shrinkage and selection operator (LASSO) Cox regression were used in sequence to screen for hematological parameters with prognostic value, and the HRM was constructed based on these parameters. The HRM was used to calculate the risk score for each patient in the training cohort and test cohort, and the optimal cutoff value of the risk score generated in the LASSO Cox regression divided patients into a high-risk group and low-risk group; the difference in PFS between the two groups was assessed using Kaplan-Meier survival analysis. The prognostic efficacy of the risk score and each hematological parameter was compared using ROC curves (method = bootstrap).

In the training cohort, other clinical prognostic factors were screened using Cox stepwise regression analysis (backward: likelihood ratio). A nomogram was formulated based on the results of multivariate Cox regression analysis. Each covariate was assigned a score range according to the Cox proportional hazard ratio; when the user inputs an exact value of the covariate into the nomogram, the score on the point scale vertically corresponding to the covariate was the risk score of the covariate, and the nomogram score was the total score obtained by summing the risk scores of the eight covariates. The Harrell’s Concordance Index (C-index) and calibration curves were used to evaluate the efficiency of prognosis. Decision curve analysis was used to assess the net benefit and net reduction of this clinical prognostic model. The stability of this model was validated in the test cohort. The correlations between the metrics that made up the nomogram were analyzed, and the differences in PFS were assessed in different subgroups.

Continuous variables are presented as the mean ± standard deviation or median values with interquartile ranges as appropriate, and categorical variables are presented as counts (with percentages). The two-tailed t-test and Mann-Whitney U-test were used to compare continuous variables as appropriate; the chi-squared test was used to compare categorical variables. A P-value <0.05 was considered to indicate a statistically significant difference. Statistical analysis and plot generation were performed using SPSS, version 26.0 (SPSS, Inc., Chicago, IL, USA) and R language, version 4.1.0 (The R Foundation for Statistical Computing, Vienna, Austria).

## Results

### Clinical Characteristics

A total of 274 patients (59.9% female and 40.1% male) with AM were included in this study. Patients’ age ranged from 20 to 95 years, and the mean age was 53.8 ± 13.4 years. A total of 104 cases (38%) of AM were located in the skull base and 170 cases (62%) in the non-skull base. A total of 204 patients (74.5%) had a KPS score ≥70, and 70 patients (25.5%) had a score <70. The mean maximum tumor size was 4.7 ± 1.6 cm. There were 137 patients (50%) with Simpson grade I, 95 patients (34.7%) with grade II, 16 patients (5.8%) with grade III, and 26 patients (9.5%) with grade IV. AM invaded the skull in 50 patients (18.2%), and 107 patients had PTBE. The mean Ki-67 index was 10.4 ± 7.7%. A total of 77 patients (28.1%) received PORT. As of June 30, 2021, 115 patients (42%) had experienced tumor progression, with a 3-year progression rate of 29.9% and a 5-year progression rate of 39.4%. The average follow-up time was 4.9 ± 1.2 years.

All patients were randomly divided into a training cohort (n = 192, 70%) and test cohort (n = 82, 30%). Clinical characteristics of the training cohort and test cohort are shown in **Table 1**. There were no significant differences in the clinical characteristics between the two cohorts (P >0.05). ROC curve analysis showed that the optimal cutoff values of HBG, NLR, PLR, LMR, RDW, FIB, DD, AGR, and LDH were 143 g/L, 1.79, 116.8, 4.15, 12.5%, 3.0 g/L, 0.06 μg/ml, 1.67, and 181 U/L, respectively. There were no significant differences in hematological parameters after dichotomization conversion between the training cohort and test cohort (P > 0.05; **Table 2**).

**Table 1:** Clinical characteristics of the training cohort and test cohort.

Characteristics	Training cohort (n = 192, 70%)	Test cohort (n = 82, 30%)	P
Gender			0.407
Female	118 (61.5%)	46 (56.1%)	
Male	74 (38.5%)	36 (43.9%)	
Age	54.0 (46.0–64.8)	53.0 (44.0–63.0)	0.974
KPS	80 (70–90)	80 (60–90)	0.185
Hypertension	45 (23.4%)	15 (18.3%)	0.346
Diabetes	13 (6.8%)	5 (6.1%)	0.837
Tumor location			0.812
Skull base	72 (37.5%)	32 (39.0%)	
Non-skull base	120 (62.5%)	50 (61.0%)	
Maximum size	4.6 (3.6–5.9)	4.5 (3.5–5.9)	0.975
Skull invasion	35 (18.2%)	15 (18.3%)	0.990
PTBE	107 (55.7%)	46 (56.1%)	0.955
EOR			0.107
GTR	165 (85.9%)	64 (78.0%)	
STR	27 (14.1%)	18 (22.0%)	
Ki-67	9 (5–19)	10 (5–15)	0.612
PORT	52 (27.1%)	25 (30.5%)	0.566
HBG	130.0 (121.0–141.0)	130.5 (120.0–142.3)	0.773
PLT	208.0 (171.0–248.0)	216.5 (173.8–251.3)	0.928
NE	4.0 (3.2–5.3)	4.1 (3.1–6.0)	0.456
LY	1.8 (1.5–2.2)	1.7 (1.3–2.1)	0.257
MO	0.47 (0.35–0.58)	0.48 (0.37–0.60)	0.453
RDW	13.3 (12.6–13.8)	13.3 (12.7–13.8)	0.581
FIB	2.84 (2.40–3.49)	2.72 (2.20–3.38)	0.085
DD	0.126 (0.070–0.224)	0.118 (0.061–0.218)	0.491
ALB	41.3 (38.8–43.5)	42.1 (39.5–44.0)	0.122
GLOB	24.9 (22.3–27.2)	25.1 (21.4–27.2)	0.742
LDH	174.5 (148.0–201.8)	169.5 (151.8–198.5)	0.961
Progression	79 (41.1%)	36 (43.9%)	0.672

KPS, Karnofsky Performance Status score; PTBE, peritumoral brain edema; EOR, extent of resection; GTR, gross total resection; STR, subtotal resection; PORT, postoperative radiotherapy; HBG, hemoglobin; PLT, platelet; NE, neutrophil; LY, lymphocyte; MO, monocyte; RDW, red blood distribution width; FIB, fibrinogen; DD, D-dimer; ALB, albumin; GLOB, globulin; LDH, lactate dehydrogenase. Reference range: HBG 115–150 g/L; PLT (125–350) *10^9/L; NE (1.8–6.3) *10^9/L; LY (1.1–3.2) *10^9/L; MO (0.1–0.6) %; RDW (1–20) %; FIB 2–4 g/L; DD 0–0.3 μg/ml; ALB 35–55 g/L; GLOB 20–35 g/L; LDH 75–245 U/L.

**Table 2:** The optimal cutoff values of hematological parameters and weighting coefficient.

Hematological parameters	Training cohort (n = 192)	Test cohort (n = 92)	P	Weighting coefficient
HBG			0.792	Exclude
≥143	44 (22.9%)	20 (24.4%)		
<143	148 (77.1%)	62 (75.6%)		
NLR			0.701	0.833
≥1.79	131 (68.2%)	54 (65.9%)		
<1.79	61 (31.8%)	28 (34.1%)		
PLR			0.267	0.583
≥116.8	96 (50.0%)	47 (57.3%)		
<116.8	96 (50.0%)	35 (42.7%)		
LMR			0.238	−0.065
≥4.15	85 (44.3%)	30 (36.6%)		
<4.15	107 (55.7%)	52 (63.4%)		
RDW			0.522	Exclude
≥12.5	165 (85.9%)	68 (82.9%)		
<12.5	27 (14.1%)	14 (17.1%)		
FIB			0.499	0.199
≥3.0	81 (42.2%)	31 (37.8%)		
<3.0	111 (57.8%)	51 (62.2%)		
DD			0.393	0.712
≥0.06	154 (80.2%)	62 (75.6%)		
<0.06	38 (19.8%)	20 (24.4%)		
AGR			0.170	−0.239
≥1.67	88 (45.8%)	45 (54.9%)		
<1.67	104 (54.2%)	37 (45.1%)		
LDH			0.345	0.623
≥181	82 (42.7%)	30 (36.6%)		
<181	110 (57.3%)	52 (63.4%)		

HBG, hemoglobin; NLR, neutrophil-to-lymphocyte ratio; PLR, platelet-to-lymphocyte ratio; LMR, lymphocyte-to-monocyte ratio; RDW, red blood distribution width; FIB, fibrinogen; DD, D-dimer; AGR, albumin-to-globulin ratio; LDH, lactate dehydrogenase. Reference range: HBG 115–150 g/L; RDW (1–20) %; FIB 2–4 g/L; DD 0–0.3 μg/ml; ALB 35–55 g/L; GLOB 20–35 g/L; LDH 75–245 U/L.

### Establishment and Validation of the Hematological Risk Model

**Figure 1:**
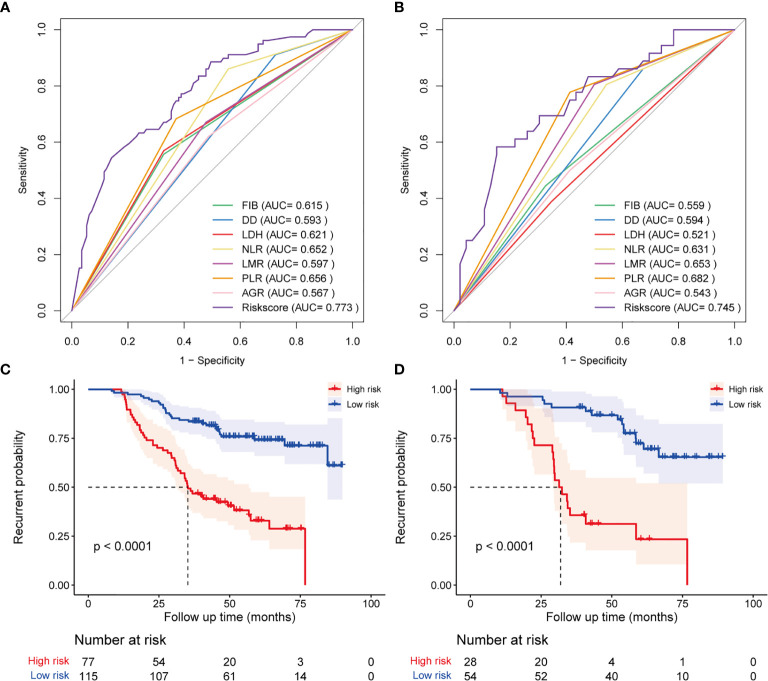
Diagnostic accuracy and prognostic performance assessment of the hematological risk model (HRM). ROC curve shows the difference in diagnostic accuracy between HRM and a single hematological parameter at the end of follow-up in the training cohort **(A)** and test cohort **(B)**. Kaplan-Meier survival analysis for progression-free survival of patients classified by hematological risk in the training cohort **(C)** and test cohort **(D)**.

In the training cohort, the correlation between each hematological parameter and PFS of patients was analyzed using univariate Cox regression. The results showed no prognostic value for HBG and RDW (P >0.05; **Supplementary Figure 1**). After excluding RDW and HBG, to screen the hematological parameters with independent prognostic value, we included the remaining hematological parameters into the LASSO Cox regression analysis and found that high levels of FIB, DD, NLR, LDH, and PLR and low levels of AGR and LMR were independent risk factors for progression. The HRM was constructed based on these parameters, and the weighting coefficient of NLR, LMR, PLR, FIB, DD, LDH, and AGR were 0.833, −0.065, 0.583, 0.199, 0.712, 0.623, and −0.239, respectively (**Table 2**). The HRM was used to calculate the hematological risk score for each patient in the training cohort and test cohort. ROC curve analysis showed that the diagnostic value of the HRM-generated risk score was higher than single hematological parameters in the training cohort [area under the curve (AUC) = 0.773, 95% confidence interval (CI) 0.707–0.839; **Figure 1A**], and the same results were observed in the test cohort (AUC = 0.745, 95% CI 0.637–0.852; **Figure 1B**). The optimal cutoff value of the risk score was 2.05, which divided patients into a high-risk group and low-risk group. In the training cohort, log-rank analysis showed that PFS in the low-risk group (mean PFS = 6.07 years, 95% CI 5.66–6.48) was longer than that in the high-risk group (mean PFS=3.64 years, 95% CI 3.17–4.11; P <0.001; **Figure 1C**); the same results were obtained in the test cohort (mean PFS = 6.11 years, 95% CI 5.58–6.65; mean PFS = 3.36 years, 95% CI 2.63–4.08; P <0.001; **Figure 1D**).

To further determine the diagnostic performance of the HRM at different times, we plotted time-dependent ROC curves. The results showed that diagnostic performance of the HRM at postoperative 3 years (AUC = 0.759, 95% CI 0.689–0.830) and 5 years (AUC = 0.786, 95% CI 0.703–0.868) was better than that at 1 year (AUC = 0.539, 95% CI 0.210–0.867) in the training cohort (**Supplementary Figure 1A**). The same results were observed in the test cohort (AUC at 1 year =0.684, 95% CI 0.464–0.905; AUC at 3 years = 0.809, 95% CI 0.702–0.915; AUC at 5 years = 0.755, 95% CI 0.630–0.879; **Supplementary Figure 1B**). We also plotted time-dependent ROC curves for a single hematological parameter from postoperative year 1 to year 5. The ROC curves indicated that the diagnostic performance of the HRM was better than that of any single hematology parameter when the time exceeded 2 years in the training cohort (**Supplementary Figure 1C**); in the test cohort, the diagnostic performance of the HRM was the best at all time points (**Supplementary Figure 1D**).

### Establishment and Validation of the Clinical Prognostic Model

To further improve the diagnostic performance and applicability of the prognostic model, we sought to identify other clinical prognostic factors and incorporate them into the model. In the training cohort, we included gender, age, KPS score, hypertension, diabetes, tumor size, location, skull invasion, PTBE, EOR, Ki-67 index, PORT, and risk score into the univariate Cox regression analysis. The results indicated that age, maximum tumor size, location, PTBE, EOR, Ki-67 index, and risk score were associated with the PFS (**Table 3**). Multivariate Cox regression analysis (backward: likelihood ratio) showed that age, location, maximum size, PTBE, EOR, Ki-67 index, PORT, and risk score were independent prognostic factors for the progression of AM. The Cox regression results of the test cohort are also shown in **Table 3**. We plotted time-dependent ROC curves for each clinical factor associated with PFS. **Supplementary Figure 2** shows the diagnostic accuracy of each factor for progression.

**Table 3:** Univariate and multivariate COX regression analysis for clinical factors.

Factors	PFS-Univariate COX analysis			PFS-Multivariate COX analysis		
(Training cohort)	HR	95% CI	P value	HR	95% CI	P value
Gender	0.800	0.500–1.27	0.335			
Age	1.02	1.01–1.04	0.014	1.02	1.00–1.04	0.100
KPS	0.993	0.980–1.01	0.351			
Hypertension	1.04	0.621–1.74	0.879			
Diabetes	1.75	0.843–3.65	0.133			
Tumor location	2.11	1.35–3.28	0.001	1.95	1.20–3.16	0.007
Maximum size	1.21	1.06–1.38	0.004	1.17	0.992–1.39	0.062
Skull invasion	0.743	0.402–1.37	0.343			
PTBE	2.40	1.47–3.90	<0.001	1.84	1.11–3.05	0.019
EOR	3.32	2.01–5.50	<0.001	4.63	2.49–8.59	<0.001
Ki-67	1.04	1.01–1.06	0.004	1.08	1.04–1.13	<0.001
PORT	1.19	0.738–1.93	0.470	0.32	0.150–0.695	0.004
Risk score	2.90	2.09–4.04	<0.001	2.37	1.70–3.29	<0.001
Factors	PFS-Univariate COX analysis			PFS-Multivariate COX analysis		
(Test cohort)	HR	95% CI	P value	HR	95% CI	P value
Gender	0.560	0.280–1.12	0.103			
Age	1.03	1.00–1.05	0.050			
KPS	0.989	0.969–1.01	0.277			
Hypertension	1.34	0.609–2.94	0.469			
Diabetes	0.328	0.045–2.40	0.272			
Tumor location	2.24	1.16–4.34	0.016	2.87	1.30–6.32	0.009
Maximum size	1.22	0.999–1.49	0.051	1.30	1.02–1.66	0.031
Skull invasion	0.788	0.327–1.90	0.594			
PTBE	2.21	1.06–4.58	0.034	3.19	1.35–7.51	0.008
EOR	2.44	1.21–4.93	0.013	9.46	2.43–36.83	0.001
Ki-67	1.07	1.03–1.10	<0.001	1.07	1.02–1.12	0.006
PORT	1.55	0.787–3.03	0.206	0.19	0.050–0.727	0.015
Risk score	2.70	1.67–4.38	<0.001	2.45	1.42–4.21	0.001

KPS, Karnofsky Performance Status score; PTBE, peritumoral brain edema; EOR, extent of resection; GTR, gross total resection; STR, subtotal resection; PORT, postoperative radiotherapy; HR, hazard ratio.

**Figure 2:**
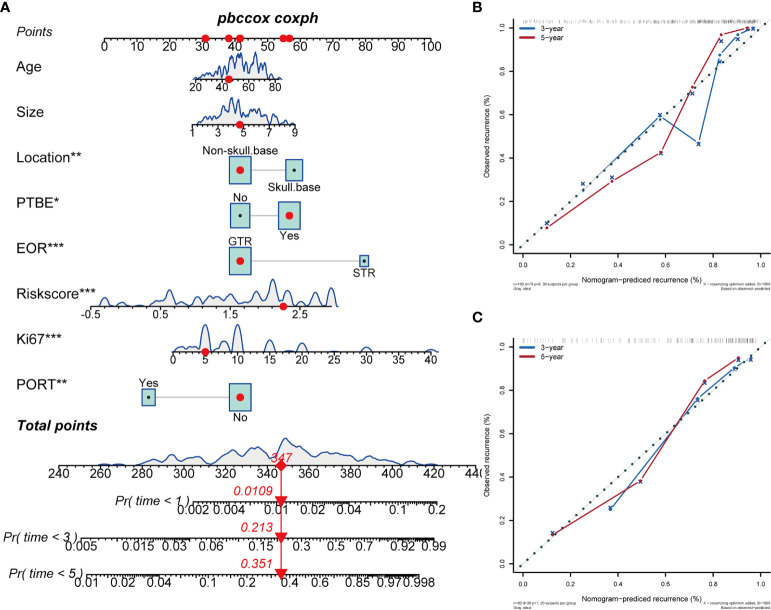
A nomogram constructed based on hematological parameters and clinical prognostic factors predicts the progression of WHO grade II meningiomas. The continuous variable curves represent the data distribution trends of continuous covariates in this study; the box area represents the proportion of categorical variables in the overall data. The nomogram score is the total score obtained by summing the risk scores of the eight covariates. The 1-year, 3-year, and 5-year tumor progression probabilities of each patient are the value on the probability scales corresponding vertically to the nomogram score. **(A)** Nomogram in WHO grade II meningiomas. **(B)** Calibration curves of the Nomogram at 3- and 5-years in the training cohort. **(C)** Calibration curves of the Nomogram at 3- and 5-years in the test cohort. *P > 0.01 and P < 0.05; **P > 0.001 and P < 0.01; ***P < 0.001.

**Figure 3:**
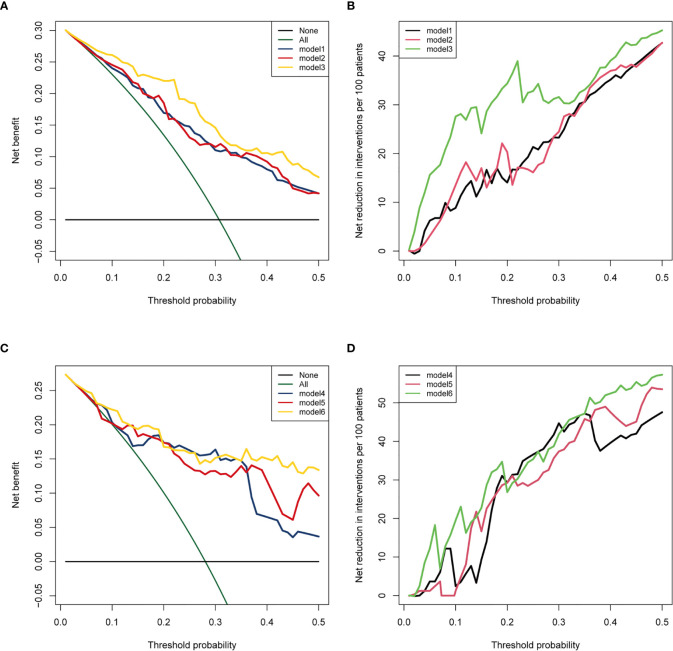
Clinical decision curve analysis demonstrates the clinical benefits of the clinical prognostic model for predicting WHO grade II meningiomas progression. **(A)** Net benefit curve in the training cohort; **(C)** Net benefit curve in the test cohort: The green line represents the net benefit of providing all patients with the intervention (such as high frequency of follow-up, postoperative radiotherapy, drug, and even biopsy), regardless of whether the tumor progression or not. Some patients without tumor progression underwent unnecessary intervention. The black line represents the net benefit of providing no patients with intervention, regardless of whether the tumor progression or not, so the net benefit was 0. The blue line (model 1 and model 4) represents the net benefit of individualized intervention after using the hematological risk model to assess the progression probability; model 2 and model 5 (red line): clinical prognostic factors; model 3 and model 6 (yellow line): clinical prognostic model. **(B)** Net reduction curve in training cohort; **(D)** Net reduction curve in test cohort: the net reduction curves reveal how many patients can avoid unnecessary intervention (such as high frequency of MRI examination) per 100 patients without missing any meningioma progression during the follow-up period, in the basis of decision derived from HRM (black line), clinical factors (brick red line), and clinical prognostic model (green line).

We established a clinical prognostic model built on the Cox regression analysis results in the training cohort and presented it as a nomogram (**Figure 2A**). Each covariate included in the nomogram was assigned a score range based on hazard ratio in the Cox regression analysis. Doctors only need to input the information of patients with AM, and the nomogram automatically calculates the total score of these eight covariates and the probability of progression at 1, 3, and 5 years. The original C-index of this nomogram was 0.81, and the corrected C-index was 0.79. The calibration curve showed favorable predictive accuracy of the nomogram at 3 and 5 years postoperatively (**Figure 2B**). To determine the stability of this nomogram, we used the data of the test cohort for validation; the original C-index was 0.81, and the corrected C-index was 0.80. The calibration curve also showed favorable predictive accuracy of the nomogram at 3 and 5 years postoperatively in the test cohort (**Figure 2C**). In addition, we performed clinical decision analysis of this nomogram to determine the clinical benefit. The net benefit curves showed that the clinical prognostic model yielded significant clinical net benefit in both the training cohort (**Figure 3A**, model 3) and test cohort (**Figure 3C**, model 6); the net reduction showed an excellent potential of the clinical prognostic model to reduce unnecessary examinations without omitting tumor progression (**Figure 3B**, model 3; **Figure 3D**, model 6).

### Subgroup Analysis

**Figure 4:**
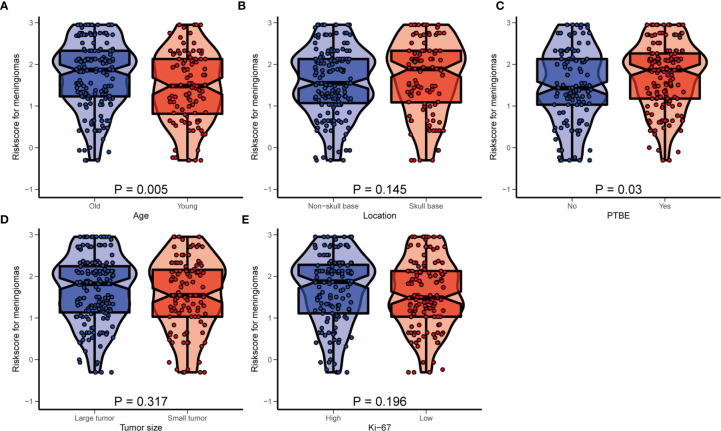
The distribution of hematological risk score in different subgroup. **(A)** Old patients ≥51 years old, young patients <51 years old; **(B)** Patients were divided into the non-skull base group and skull group according to the tumor location. **(C)** PTBE, peritumoral edema; **(D)** large tumor: maximum size ≥4.2 cm, small tumor: maximum size <4.2 cm; **(E)** high Ki-67 index: ≥10%, low Ki-67 index: <10%.

To further verify the stability and applicability of the HRM, we divided the patients into different subgroups on the basis of prognostic factors included in the nomogram and compared the differences in risk score and the differences in prognostic performance of the HRM among the different subgroups. We used ROC curves to derive optimal cutoff values for age (51 years, AUC=0.597, 95% CI 1.17–3.26), tumor size (4.2 cm, AUC=0.638, 95% CI 1.65–4.70), and Ki-67 index (10%, AUC=0.659, 95% CI 1.55–4.17). According to the above optimal cutoff values, patients were divided into old (62.4%) and young (37.6%) groups, large tumor (61.3%) and small tumor (38.7%) groups, and high Ki-67 index (51.1%) and low Ki-67 index (48.9%) groups. Patients were divided into skull base and non-skull base groups according to the tumor location, and into PTBE and non-PTBE groups according to the presence of peritumoral edema. Box violin plots showed that the risk score was higher in the old group (*vs.* young) and PTBE group (P = 0.005, P = 0.030; **Figures 4A, C**). There were no significant differences in risk scores between the different tumor locations, tumor size, and Ki-67 index groups (P > 0.05; **Figures 4B, D, E**).

**Figure 5:**
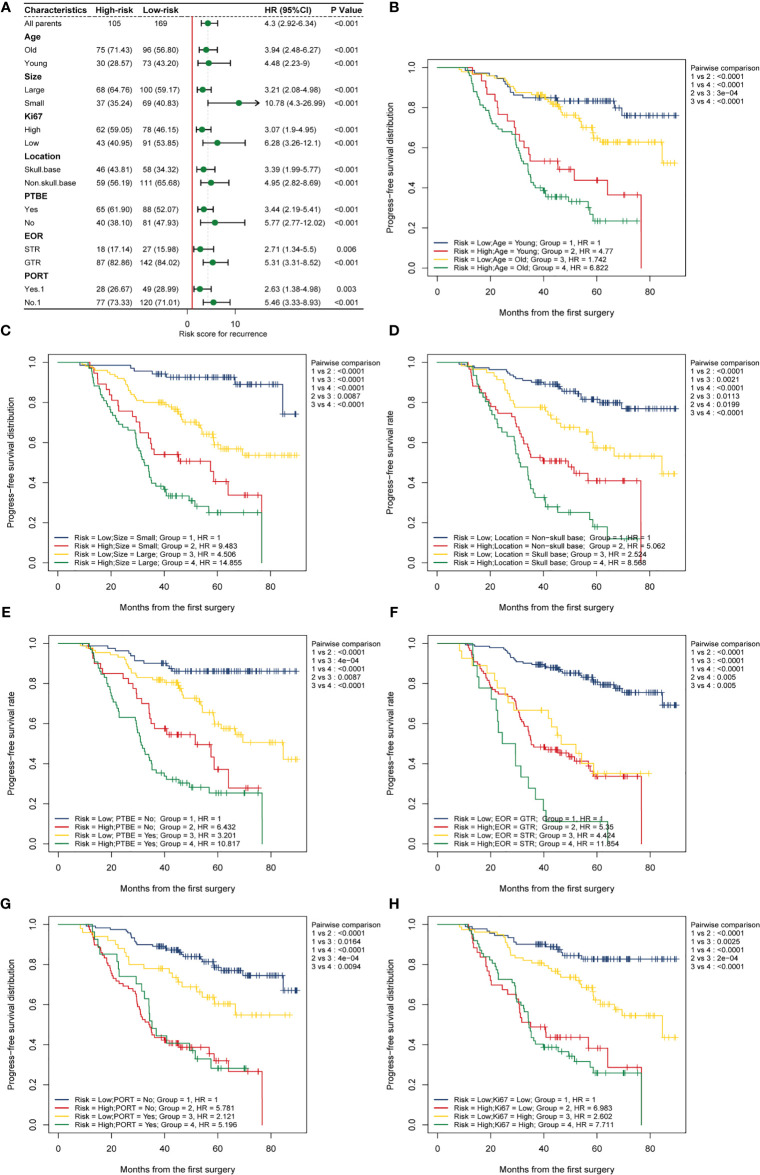
Subgroup analysis. **(A)** Subgroup analysis forest map shows the prognostic value of the hematological risk model in different subgroups categorized by age, location, tumor size, PTBE, EOR, Ki-67, and PORT. A high risk score is an independent risk factor for meningioma progression in different subgroups. **(B–H)** Two-factors Kaplan-Meier curves of progress-free survival for patients with AM categorized by risk scores and other clinical factors: **(B)** age; **(C)** size; **(D)** tumor location; **(E)** PTBE, peritumoral brain edema; **(F)** EOR, extent of resection; STR, subtotal resection; GTR, gross total resection; **(G)** PORT, postoperative radiotherapy; **(H)** Ki-67 index.

Considering the potential heterogeneity among the enrolled patients, we compared the differences in prognostic performance of the HRM on PFS in different subgroups. Encouragingly, subgroup analysis forest maps suggested that the hematological risk score generated by the HRM was an independent risk factor for the progression of AM in different subgroups, regardless of age, tumor size, tumor location, presence of PTBE, EOR, Ki-67 index level, or PORT (HR > 1, P < 0.05; **Figure 5A**), which further reflected the excellent stability and universality of the HRM. In parallel, we also investigated the ability of the HRM to predict the PFS of patients in different subgroups. Kaplan-Meier survival curves showed that a high hematologic risk score increased the postoperative progression rate of AM and diminished PFS, regardless of age, tumor size, tumor location, presence of PTBE, EOR, Ki-67 index level, or PORT (**Figures 5B–H**, subgroup 1 *vs.* subgroup 2, subgroup 3 *vs.* subgroup 4; P < 0.05). In the hematological low-risk group, large tumor size, PTBE, and high Ki-67 index were associated with higher relapse probability and shorter PFS (**Figures 5C, E, H**, subgroup 1 *vs.* subgroup 3; P < 0.05); nevertheless, in the high-risk group, these factors did not apparently affect the progression risk of AM or PFS of patients (subgroup 2 *vs.* subgroup 4; P > 0.05). The PFS was shorter in the high-risk group than that in the low-risk group, regardless of tumor location and EOR (**Figures 5D, F**, subgroup 1 *vs.* subgroup 2, subgroup 3 *vs.* subgroup 4; P < 0.05). The PFS was shorter in patients with tumors located at the base of the skull and STR, regardless of hematological risk (**Figures 5D, F**, subgroup 1 *vs.* subgroup 3, subgroup 2 *vs.* subgroup 4; P < 0.05). When performing the two-factor subgroup analysis of hematological risk and PORT, we found that PORT increased the progression probability of AM in the hematological low-risk group (**Figure 5G**, subgroup 1 *vs.* subgroup 3; HR = 2.12; P = 0.016). In consideration of the possible interaction between the effects of PORT and EOR, we specifically compared the effect of PORT on PFS in patients with different EORs. We observed a significantly improved PFS with adjuvant radiotherapy compared with no adjuvant radiotherapy after STR (**Supplementary Figure 3**, subgroup 2 *vs.* subgroup 4; P < 0.05). In patients who underwent GTR, adjuvant radiotherapy did not improve PFS (subgroup 1 *vs.* subgroup 3; P > 0.05).

## Discussion

Meningiomas are the most common primary intracranial tumors and arise from the arachnoid cap cells of the dura mater (16). Although most meningiomas are benign (WHO grade I, 80.5%), AMs (WHO grade II, 17.7%) are more aggressive with a tendency for relapse (17). The WHO classification is an important prognostic tool that is based on histological criteria, but cannot fully predict tumor progression. There is substantial variation within the same grade among tumors, with studies reporting indolent behavior with no progression in up to 71% of AM cases (3). Therefore, it is especially important to accurately predict tumor progression and realize hierarchical tumor management. Growing evidence in recent decades has shown that genetic alterations, epigenetic modifications, and modifications in histones play a crucial role in tumorigenesis and the progression of meningiomas (18). However, most of these individualized medical treatments based on gene detection are invasive and expensive and cannot fully define the spatial heterogeneity of tumors. Numerous studies have demonstrated that hematological parameters have great potential in tumor prognostic stratification (7, 9, 19–21); unlike genetic markers, hematological parameters obtained from preoperative peripheral blood tests have the advantages of non-invasiveness, low cost, and easy access. Previous studies have demonstrated that high preoperative levels of NLR and FIB and low levels of LMR are associated with adverse clinical outcomes of AM (7, 11, 22). However, these studies have included too few hematological parameters, and, by default, each hematological parameter has the same weighting coefficient to predict the progression of AM; whether some hematological parameters with prognostic value in other tumors have the same effect on AM has not been explored. Based on the complexity of the tumor microenvironment, it remains to be seen whether the prognostic models proposed by these studies can fully reflect tumor characteristics and predict tumor progression. In the present study, we extensively collected hematological parameters, including NLR, PLR, LMR, DD, FIB, LDH, AGR, RDW, and HBG. The relationship of each hematological parameter with the progression of AM was assessed, and the HRM was constructed based on the parameters that were found to have prognostic value. The HRM exhibited moderate prognostic performance, with better long-term prediction (AUC = 0.773 in the training cohort; AUC = 0.745 in the test cohort). To further improve the predictive power and applicability of the HRM, we constructed a clinical prognosis model by incorporating clinical prognosis factors into the HRM, and presented it in the form of nomogram; the corrected C-index of nomogram was 0.79. To the best of our knowledge, this is the first prognostic model of AM progression based on multiple hematological parameters and multiple clinical prognostic factors, that has a favorable prediction accuracy.

### Hematological Parameters

A growing number of studies have suggested that chronic inflammation participates in the occurrence, invasion, and metastasis of tumors (8, 9, 11). In the past few decades, the central nervous system has been considered immune-privileged due to the existence of blood-brain barrier. Recently, infiltrative inflammatory cells have been found to be a key component of the neurological tumor microenvironment. Hypoxia and abnormal vascular proliferation occur owing to excessive tumor growth that leads to the infiltration of various immune cells, such as neutrophils, lymphocytes, and macrophages (23). The degradation of tight junctions between endothelial cells and the massive secretion of inflammatory substances contributes to significant destruction of the blood-brain barrier; peripheral immune cells break through the blood-brain barrier to converge and enrich at the site of the tumor, which further aggravates the infiltration of inflammatory cells in the tumor microenvironment (24). The infiltration of monocytes and neutrophils in the tumor microenvironment can lead to abnormal vascular proliferation and immunosuppressive factor production, thereby avoiding being killed by lymphocytes and achieving tumor cell metastasis (23). Lymphocytes play an important role in antitumor immunity by secreting cytokines or differentiating into cytotoxic T cells to achieve tumor cytotoxic death (25, 26). Platelets secrete angiogenic factors to influence the tumor microenvironment (27). In this study, the weighting coefficients of NLR, LMR, and PLR in the HRM were 0.833, −0.065, and 0.583, which further verified the previous conclusions. Kuranari et al. demonstrated that a NLR ≥ 2.6 was associated with a short PFS in patients with meningiomas (22). Liang et al. demonstrated that an LMR < 4.78 was an independent risk factor for poor outcomes (7). Their optimal cutoff values were higher than those we obtained (NLR 1.78, LMR 4.15); we believe that the high progression rate caused a lower optimal cutoff value as a consequence of the exclusion of WHO grade I meningiomas in our study.

An increasing number of studies have indicated that tumorigenesis is closely associated with abnormalities in the coagulation cascade response in the early stage of tumor. The coagulation system is an integral component of the unique vascular microenvironment for tumor proliferation and progress (28, 29). FIB and DD, as important factors in the coagulation cascade and fibrinolysis processes, respectively, have been shown to be involved in the invasive process of a variety of malignancies (8, 19, 30). The “net” constructed by FIB in the extracellular matrix promotes cell adhesion and tumor invasion (30); in addition, FIB ultimately promotes angiogenesis and tumor growth by binding to growth factors such as vascular endothelial growth factor and fibroblast growth factor-2 (31, 32). Previous studies have found that a high level of DD induces the progression of oral squamous cell carcinoma, cervical cancer, and glioma (13, 19, 33). In our study, the AUCs of FIB and DD for the diagnosis of meningioma progression were 0.615 and 0.593 in the training cohort, respectively, and their weighting coefficients in the HRM were 0.199 and 0.712, respectively; this also confirmed that high levels of FIB and DD were risk factors for the progression of AM. To the best of our knowledge, this is the first study to explore the prognostic value of DD in AM.

LDH is a cytoplasmic enzyme expressed in various tissues of the whole body and involved in glycolysis. Elevated LDH levels appear to indicate an aggressive tumor phenotype (34). Under the condition of excessive metabolism of tumor cells, LDH promotes the tumor-specific Warburg effect; this produces energy and lactic acid through glycolysis, which is involved in the tricarboxylic acid cycle after being re-ingested by cancer cells (34). Wang et al. and Frank et al. have also found that LDH can recruit and regulate tumor-associated macrophage metabolism in breast cancer to affect breast cancer cell proliferation and invasion (20, 35). Ruiz-Rodado et al. demonstrated that the glioma IDH1-mut invasive cell line obtained a high glycolysis phenotype through the specific deletion of methylation of the LDH promoter, which was associated with a worse survival rate (36). At present, the mechanism by which LDH affects the metabolism and invasion of meningioma is still unclear, and further research is needed to clarify this. Previous studies have confirmed that the reduction of AGR is related to a poor prognosis in patients with glioma, that the correlation between AGR and cancer may be due to the antioxidant effect of albumin on carcinogens, such as nitrosamines and aflatoxins, and that globulin promotes the progression and metastasis of tumors (12). The HRM we constructed confirmed that a high level of LDH and a low level of AGR were associated with adverse outcomes of AM, providing more reference parameters for the prognosis assessment of meningiomas. The mechanism underlying their impact on the prognosis of meningiomas has yet to be explored.

### Clinical Prognostic Factors

In addition to hematological parameters, we also evaluated the prognostic value of other clinical factors on the progression of AM. A recent large retrospective study that enrolled 2,358 patients with AM from China showed that patients over 67.5 years old had an increased risk of mortality compared with patients less than 67.5 years old (37). Barthelemy et al. recommended hierarchical management for patients aged <55 and >75 years with AM (38). Our study indicates that clinicians should focus on patients more than 51 years due to their high progression risk. A larger tumor maximum size was found to be a hazard factor for AM progression in our study. One possible reason for this finding is that the greater mass effect made GTR more difficult (37). Univariate and multivariate Cox regression analyses showed that a tumor located at the skull base was an independent risk factor for AM progression. Considering the complex anatomy of the skull base and the limited surgical field of view, tumors located in the skull base tend to undergo subtotal resection. Our conclusion supports results from Champeaux et al., who reported that tumor location was independently associated with overall survival (39).

The PTBE is a common complication of meningioma. Various potential risk factors contribute to the formation of PTBE, such as tumor-brain barrier disruption, tumor size, location, tumor margin shape, AQP4/TRPV4 channel co-expression, and vascular endothelial and lymphatic dysfunction (40–42). Recent studies have confirmed that PTBE is associated with the WHO classification and invasiveness of meningiomas (43, 44). The SKALE scoring decision-making system, which includes PTBE and other parameters, has been demonstrated to be related to postoperative progression rate of meningiomas (45). Our study also confirmed that PTBE was an independent risk factor for the progression of AM. However, some work has found that PTBE does not lead to worse clinical outcomes (11). The Ki-67 index is a typical immunohistochemical marker for tumor cell proliferation and is widely used to assess the invasiveness of tumors in the central nervous system. It is still controversial as to whether the Ki-67 index is adequate for predicting the progression of meningioma, as is its optimal cutoff value. Several previous studies have shown that the Ki-67 index is a significant independent predictor of meningioma progression (22, 46, 47). A prospective study enrolled 159 patients with meningiomas from Sweden concluded that the Ki-67 index was a marker for time to progression rather than a predictor of progression (48). A recent meta-analysis including 5,012 patients with meningiomas found that Ki-67 index was significantly associated with PFS with a tentatively proposed cutoff of 4% (49). In our study, Cox regression analysis revealed that a high Ki-67 index was an independent risk factor for progression in the training cohort (HR = 1.08, 95% CI 1.04–1.13; P < 0.001), and the subgroup analysis showed that PFS was shorter in patients with a Ki-67 ≥10%. The difference in optimal cutoff values is due to the heterogeneity of included patients (WHO grade, age, race, etc.). In the future, the predictive value of Ki-67 and its optimal cutoff value should be better explored in a large-sample prospective study.

Currently, Simpson’s removal grade is a parameter that is widely used by neurosurgeons in clinical practice to evaluate the progression risk for meningiomas. Numerous studies have concluded that STR (Simpson grade ≥3) induces early progression of meningiomas (15, 16, 37), and our results are consistent with this finding. Considering the efficacy and neurotoxicity of radiotherapy, it is still controversial as to whether PORT is necessary as a routine treatment for AM. Based on the existing evidence, PORT is recommended for AM with STR, especially for skull base meningiomas (3, 50). However, the role of PORT in patients with completely resected AM remains undefined, and there is still no unified consensus on whether these patients need radiotherapy, or the dose of radiotherapy (17). A recent large-scale retrospective study in the United States that was based on the clinical surveillance resource oncology dataset observed significantly improved overall survival with adjuvant radiotherapy compared with no adjuvant radiotherapy after STR; in patients who underwent GTR, adjuvant radiotherapy did not improve overall survival (51). In our study, multivariate Cox regression analysis showed that PORT was an independent risk factor for progression. When performing the two-factor subgroup analysis of hematological risk and PORT, we found that PORT increased the progression probability of AM in the hematological low-risk group. This may be due to doctors’ preference to treat AM with STR and a high Ki-67 index with adjuvant radiotherapy, while the residual tumor itself may be more aggressive. Kaplan-Meier subgroup analysis showed that postoperative radiotherapy could reduce the risk of tumor progression and prolong PFS in the STR group; however, in the GTR group, PORT was not related to the PFS of patients (P > 0.05). However, some neurosurgeons and radiologists believe that PORT can reduce the progression risk and tumor burden of patients and prolong the progression time after GTR (52, 53). There is a lack of high-quality evidence for the efficacy of adjuvant radiotherapy in patients with completely resected AM, and multiple prospective studies with large samples and long-term follow-up are needed to explore the effects of adjuvant radiotherapy on PFS and overall survival at different stages in patients with GTR AMs.

### Limitations

Several limitations of this study should be acknowledged. First, this was a retrospective study carried out by a single institution, which may have introduced selection bias. Second, only hematological parameters with prognostic value in other tumors were included in this study, and other hematological parameters with prognostic value for meningioma progression may have been omitted. Third, molecular markers such as NF2 and TERT were not included in our study because some patients had incomplete immunohistochemistry analysis or gene sequencing. Finally, we normalized the hematological indexes, and their optimal cutoff values remain to be verified in future large-sample prospective studies. The prognostic impact of continuous changes in hematological indexes also needs further exploration.

## Conclusions

Our study confirmed the prognostic value of preoperative hematological parameters in patients with WHO grade II meningiomas. The constructed HRM is an independent prognostic factor for the PFS of patients. Age, tumor location, tumor size, PTBE, EOR, Ki-67, and PORT were also related to the prognosis of patients. The nomograph constructed based on the HRM has a favorable prediction performance and could be used to obtain good clinical benefits. A large-scale prospective study is required in the future to confirm the stability and accuracy of our clinical prognostic model.

## Data Availability Statement

The raw data supporting the conclusions of this article will be made available by the authors, without undue reservation.

## Author Contributions

PG, DY, and XL contributed to the conceptualization and methodology of this study. PG, TK, XZ, and YZ contributed to data curation. PG, SY, DZ, and HJ performed the data analysis. PG, HL and DC wrote the original draft. DY and XL contributed to draft revision. All authors agree to be accountable for the content of the work. All authors contributed to the article and approved the submitted version.

## Funding

This research is supported by the Henan Natural Science Fund Project, China (no. 212300410401).

## Conflict of Interest

The authors declare that the research was conducted in the absence of any commercial or financial relationships that could be construed as a potential conflict of interest.

## Publisher’s Note

All claims expressed in this article are solely those of the authors and do not necessarily represent those of their affiliated organizations, or those of the publisher, the editors and the reviewers. Any product that may be evaluated in this article, or claim that may be made by its manufacturer, is not guaranteed or endorsed by the publisher.

## Supplementary Material

Supplementary Table 1 Univariate COX regression analysis for hematological parameters.

Supplementary Figure 1 Time-dependent ROC cures of hematological parameters.

Supplementary Figure 2 Time-dependent ROC curves for predictive factors in the training cohort and test cohort.

Supplementary Figure 3 Kaplan-Meier analysis curve for patients with atypical meningiomas categorized by extent of resection and postoperative radiotherapy.
