# Supplementary material for: A Clinical Prognostic Model Based on Preoperative Hematological and Clinical Parameters Predicts the Progression of Primary WHO Grade II Meningioma
Source: Front Oncol. 2021 Oct 11;11:748586. doi: 10.3389/fonc.2021.748586 (PMC8542933; doi:10.3389/fonc.2021.748586)
Supplement: Supplementary Table 1 — Univariate COX regression analysis for hematological parameters. [file DataSheet_1.docx]

Supplementary Material

## Supplementary Figures

**Supplementary Figure 1.** (A) The AUCs of HRM at 1-, 3- and 5-years in training cohort; (B) The AUCs of HRM at 1-, 3- and 5-years in training cohort and in test cohort. Comparison of the time-dependent ROC curves of between risk score generated by HRM and a single hematological parameter for postoperative 1 year to 5 years in training cohort (C) and in test cohort (D).

**Supplementary Figure 2.** (A) Time-dependent ROC curves for risk score and clinical factors in the training cohort. The prognostic accuracy of the risk index is optimal when the time exceeds approximately 2.3 years postoperatively. (B) Time-dependent ROC curves for risk score and clinical factors in the test cohort. The prognostic accuracy of the risk index is optimal at any time.

**Supplement Figure 3.** Kaplan-Meier survival curve of progress-free survival for patients with AM categorized by extent of resection (EOR) and postoperative radiotherapy (PORT). Our study observed significantly improved PFS with adjuvant radiotherapy compared with no adjuvant radiotherapy after STR (subgroup 2 vs. subgroup 4), in patients who underwent GTR, adjuvant radiotherapy did not improve PFS (subgroup 1 vs. subgroup 3).

## Supplementary Tables

Supplementary table 1. Univariate COX regression analysis for hematological parameters

| Hematological parameters | Hazard ratio | 95% CI | P |
| --- | --- | --- | --- |
| NLR | 3.68 | 1.94-6.96 | **<0.01** |
| LMR | 0.488 | 0.304-0.786 | **<0.01** |
| PLR | 2.89 | 1.79-4.67 | **<0.01** |
| DD | 3.17 | 1.45-6.89 | **<0.01** |
| FIB | 2.09 | 1.34-3.27 | **<0.01** |
| LDH | 2.19 | 1.40-3.42 | **<0.01** |
| AGR | 0.622 | 0.394-0.980 | **0.040** |
| HBG | 0.755 | 0.430-1.33 | 0.329 |
| RDW | 1.73 | 0.831-3.60 | 0.143 |

HBG, hemoglobin; NLR, neutrophil-to-lymphocyte ratio; PLR, platelet-to-lymphocyte ratio; LMR, lymphocyte-to-monocyte ratio; RDW, red blood distribution width; FIB, fibrinogen; DD, D-dimer; AGR, albumin-to-globulin ratio; LDH, lactate dehydrogenase.
