# Supplementary figures and images for: A Clinical Prognostic Model Based on Preoperative Hematological and Clinical Parameters Predicts the Progression of Primary WHO Grade II Meningioma
Source: Front Oncol. 2021 Oct 11;11:748586. doi: 10.3389/fonc.2021.748586 (PMC8542933; doi:10.3389/fonc.2021.748586)

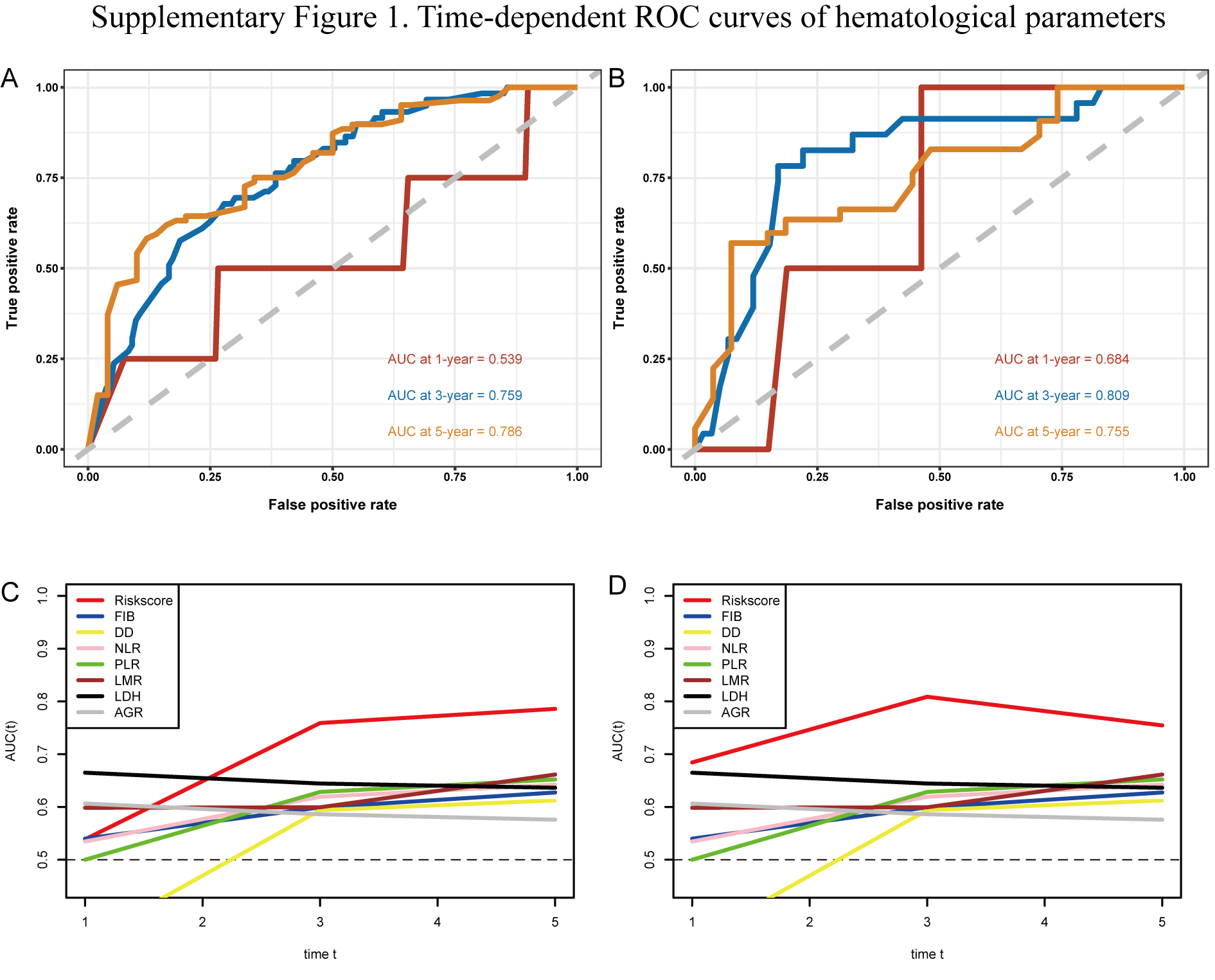

Supplement: Supplementary Figure 1 — Time-dependent ROC cures of hematological parameters. [file Image_1.tif]

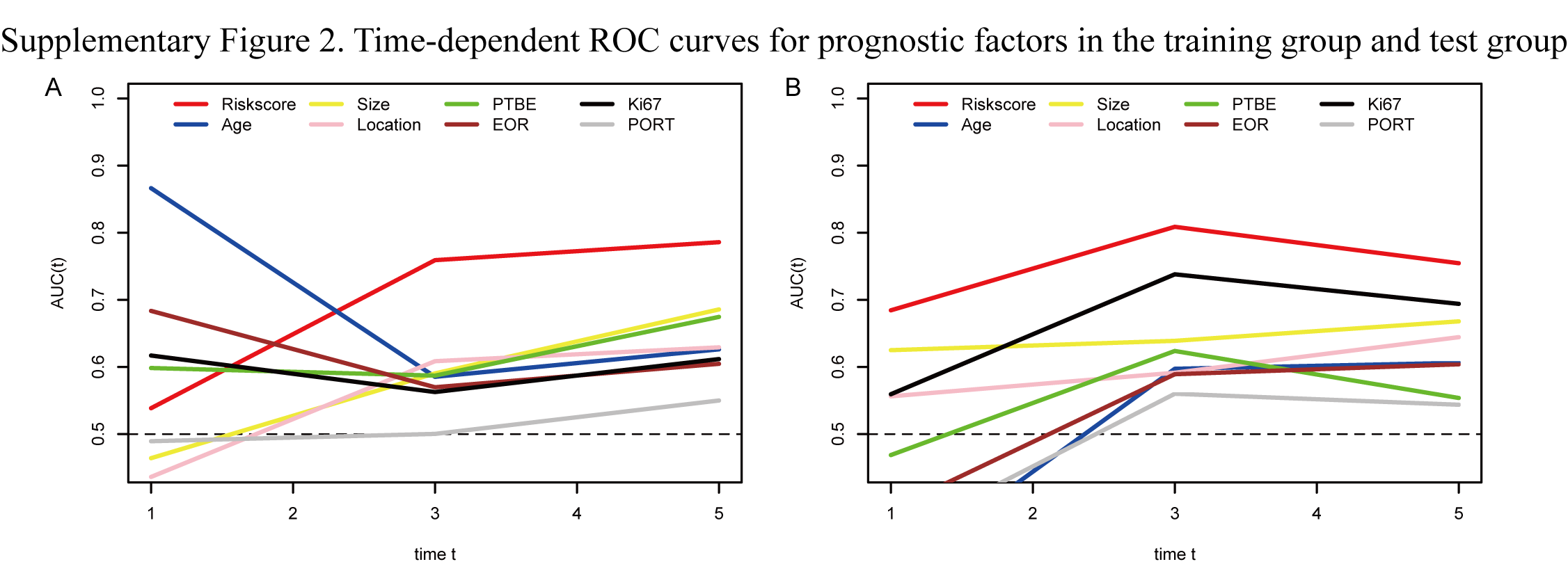

Supplement: Supplementary Figure 2 — Time-dependent ROC curves for predictive factors in the training cohort and test cohort. [file Image_2.tif]

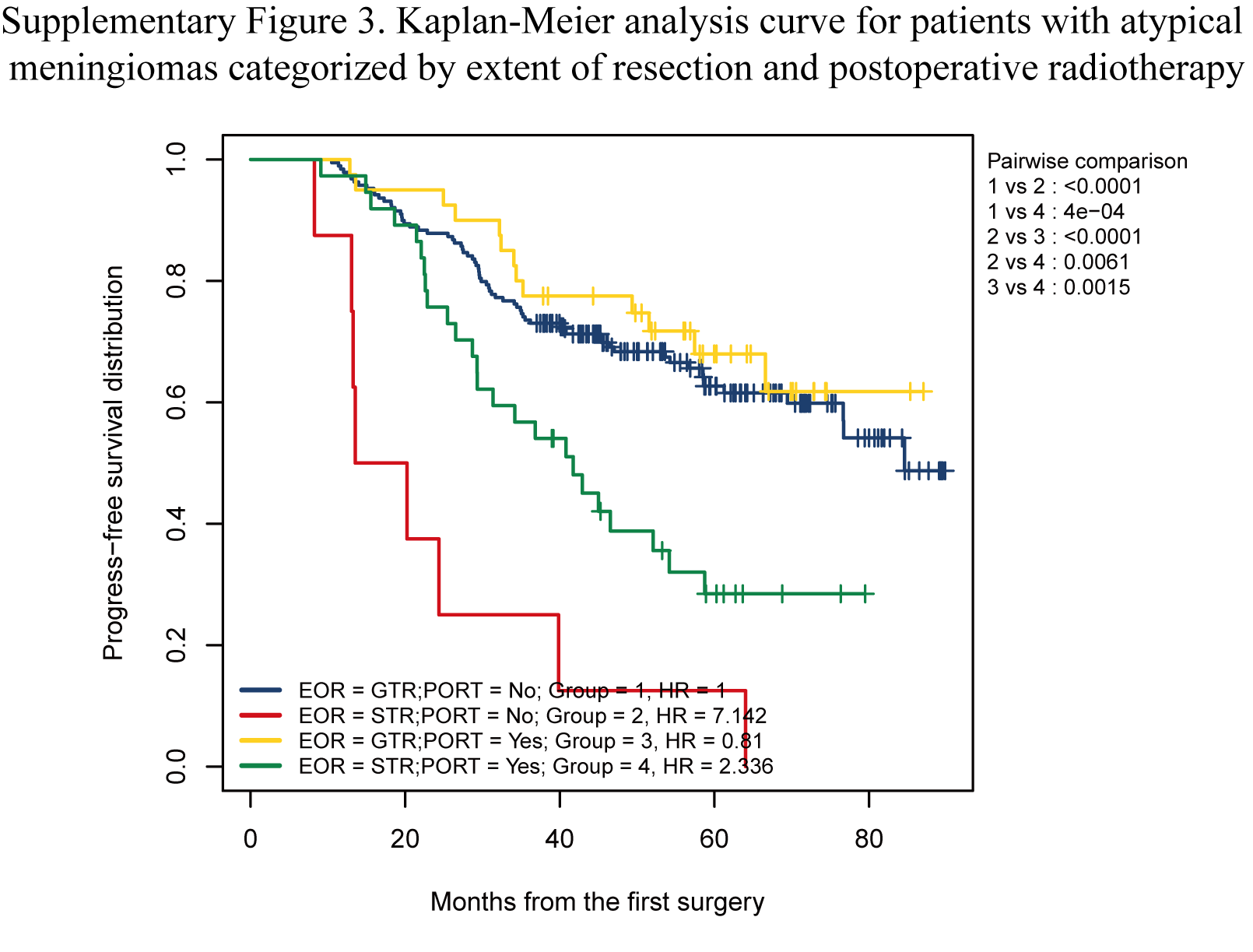

Supplement: Supplementary Figure 3 — Kaplan-Meier analysis curve for patients with atypical meningiomas categorized by extent of resection and postoperative radiotherapy. [file Image_3.tif]
